# Robbing Peter to Pay Paul

**Published:** 1952-01

**Authors:** Walter Woolley

**Affiliations:** Secretary, Bristol Medical Committee 239 Cranbrook Road, Bristol 6.


### Correspondence

### Robbing Peter to pay Paul

SIR,

Nurses in our hospitals deserve all the comfort they can be given but the turning of Brislington House into a Nurses Home by the Board of Governors, with the resultant loss of about one hundred beds for mental cases, makes one wonder if liaison between different bodies which run the Health Service is as efficient as it should be.

The Annual Report of the Board of Control states, " In previous reports we have drawn attention to the serious shortage of accommodation. There seems to be little prospect of this shortage being relieved to any great extent, though the attention of the Regional Hospital Boards has been specially drawn to the need for providing more beds." Were the Regional Board, or the Local Authority, which is responsible for the Mental Health Services, consulted or were the Local Executive Council, the Medical Committee, the Authorised Officers or the District Nurses asked for their views before this decision was taken which will increase the difficulties facing them in accommodating and treating these patients?

When will Planners learn that the closest consultations should be maintained between all interested parties from the earliest stages if best use is to be made of the inadequate existing facilities within the Health Service?

WALTER WOOLLEY

Secretary,
Bristol Medical Committee
239 Cranbrook Road,
Bristol 6.

